# Abundance of Tumor-Infiltrating B Cells in Human Epithelial Malignancies

**DOI:** 10.32607/actanaturae.27353

**Published:** 2024

**Authors:** E. A. Petrov, D. M. Malabuiok, H. Zheng, Yu. A. Mokrushina, V. A. Abrikosova, Yu. B. Kuzmin, P. V. Tzarapaev, S. O. Kochkina, I. V. Eltsov, V. D. Knorre, I. V. Smirnov, S. S. Terekhov, Z. Mamedli, N. E. Kushlinskii, D. V. Rogozhin, V. B. Matveev, P. V. Kononets, I. S. Stilidi, H. Zhang, A. G. Gabibov

**Affiliations:** Shemyakin–Ovchinnikov Institute of Bioorganic Chemistry, Moscow, 117997 Russian Federation; State Key Laboratory of Medicinal Chemical Biology and College of Life Sciences, Nankai University, 94 Weijin Road, Tianjin, 300071 China; Department of Chemistry, Lomonosov Moscow State University, Moscow, 119991 Russian Federation; Blokhin National Medical Research Center of Oncology, Moscow, 115522 Russian Federation; Endocrinology Research Center, Moscow, 117036 Russian Federation

**Keywords:** tumor-infiltrating B cells, colorectal cancer, solid tumors

## Abstract

Cancer is a major global health problem. The type of malignant neoplasm and the potency of the immune response against tumors are two of the key factors influencing the outcome of the disease. The degree of tumor infiltration by lymphocytes plays an important role in antitumor response development, generally correlating with a favorable prognosis of treatment for certain cancers. We analyzed the abundance of tumor-infiltrating B cells (TIBs) in solid tumors of different cancers. TIBs were shown to be more abundant in colon and sigmoid colon cancer samples compared with cecal, rectal, and kidney cancer samples. The median and interquartile range of the TIB fraction were 11.5% and 4–20% in colon cancer, 6% and 3–11% in sigmoid colon cancer, 2.7% and 0.7–3.7% in cecal cancer, 2.5% and 0.9–3.6% in rectal cancer, 1.4% and 1.0–2.3% in kidney cancer, and 3.0% and 1.8–12% in lung cancer, respectively. However, there were no significant differences in the abundance of TIBs among samples at different stages of the cancer. Hence, investigation of the B cell response in colon cancer is of particular interest, since increased quantities of TIBs may indicate the existence of immunogenic tumor markers or the cell-cell interactions involved in disease progression. We believe that studying the diversity of TIBs in colon cancer will increaseour understanding of the mechanisms of the disease, contributing to the identification of new molecular targets for targeted oncotherapy.

## INTRODUCTION

Malignant neoplasms are among the most challenging medical and social problems. According to the WHO, the incidence of cancers continues to increase. The most common oncological diseases are breast, lung, colorectal, and prostate cancers. Despite improvements in the treatment. as well as new therapies, the mortality rate from some types of tumors, such as lung, colorectal, and liver cancers, remains very high [1].

Tumor-infiltrating immune cells play a key role in the development of the body’s immune response to the tumor; they are capable of exhibiting both protumor and anti-tumor activity. Tumor-infiltrating immune cells are a heterogeneous set that includes T cells, B cells, natural killer (NK) cells, macrophages, neutrophils, and dendritic cells. The subset composition and percentage of tumor-infiltrating immune cells can vary depending on the type and stage of a cancer, as well as from patient to patient [2]. Tumorinfiltrating lymphocytes (TILs) involve T and B cells that have moved from the bloodstream and migrated into the tumor. The presence of TILs in a tumor may be a prognostic marker of a favorable course of the disease and the efficacy of therapy [3].

Investigation of adaptive immunity in oncoimmunology is largely focused on CD8^+^ cytotoxic T lymphocytes (CTLs). CTLs are considered to be the main effectors of the antitumor immune response; they directly destroy transformed cells. High levels of tumor infiltration by CTLs correlate with a favorable prognosis for the course of the disease and increased overall survival chances in patients with different cancers [4]. CD4^+^ T cells are an integral part of adaptive immunity, but their role in the immune response to a tumor remains unsettled [5]. Along with CD8^+^ T cells, there are tumor-specific CD4^+^ T helper (Th) cells that can recognize tumor antigens and effectively slow tumor growth in animal models in the absence of CTLs [6]. However, the bulk of the antitumor effect of CD4^+^ Th cells is the Th-mediated activation of CTLs to recognize and destroy tumor cells or activate other immune cells, in particular the B cell component of the immune response [7]. On the contrary, the subset of CD4^+^ T regulatory (Treg) lymphocytes is known to exert an immunosuppressive effect, mainly due to the production of cytokines (IL-10 and TGFβ), and suppress the antitumor function of the effector cells of the tumor microenvironment, contributing thus to malignant growth and an unfavorable outcome [8].

Less is known about B cells that infiltrate the tumor and often co-localize with T cells, sometimes forming organized lymphoid structures [9]. Tumorinfiltrating B cells (TIBs) affect malignancies through two opposing mechanisms and can both promote and suppress tumors [10]. The antitumor effects of B cells are mediated through various pathways. Upon humoral responses to tumor neoantigens, B cells differentiate into plasma cells and secrete tumor-specific antibodies that mediate antibody-dependent cellular cytotoxicity (ADCC), complement-dependent cytotoxicity (CDC), or antibody-dependent cellular phagocytosis (ADCP). In addition to antibody production, B cells can secrete a variety of cytokines, thus influencing the function of other immune cells in the tumor microenvironment in multiple directions. For example, secretion of IL-12 by B cells mediates the proliferation and antitumor action of T and NK cells and secretion of IL-10 by regulatory B cells (Breg), through suppression of the autoimmune response, has a protumor effect [11]. Also, B cells can act as antigen-presenting cells (APCs); when Th2 cells are activated by the CD40 ligand, B cells express chemokines and costimulatory factors and induce a T cell antitumor immune response [12]. Thus, TIBs have a broad potential for tumor cell destruction and exert a significant impact on the balance of activation or suppression of other immune cells in the tumor’s microenvironment.

TIBs have been studied particularly widely in breast cancer, where they are found in 25% of cases and account for up to 40% of the tumor-infiltrating lymphocyte population. In breast cancer, the prognosis of patient survival and the choice of therapy depend on the abundance of TIBs [13]. At present, the abundance of TIBs is known to positively correlate with a favorable clinical outcome of melanoma [14], ovarian cancer, non-small cell lung cancer [15], and squamous cell cervical cancer [16, 17].

The aim of this study was to assess the abundance of B lymphocytes in various nosological forms of oncological diseases and to assess the association between the B lymphocyte content and the clinical and morphological characteristics of these diseases.

## EXPERIMENTAL

The study included 50 patients undergoing surgical treatment at the Blokhin National Medical Research Center of Oncology of the Ministry of Health of the Russian Federation. The malignant nature of the tumors in all the patients was clinically verified upon routine pathomorphological examination. The study included donors who had not undergone chemotherapy before surgery. All patients gave informed consent to participate in the study. The study was conducted in compliance with current legal and ethical standards.

**Tumor cell isolation**

A ~0.5–2 cm^3^ tumor fragment was placed in a 50 mL tube with phosphate-buffered saline (PBS) immediately after tumor resection; further manipulations with the material were started within 1.5–3 h after surgery. Biological material was transported to the laboratory at room temperature. A sample was precipitated in a benchtop centrifuge (Eppendorf, Germany) for 5 min (100 g, 24°C). The supernatant was decanted, and 5 mL of the handling medium (a 1 : 1 mixture of DMEM F12 and RPMI 1640 (PanEco, Russia), 10% HyClone bovine fetal serum (Cytiva, USA), and an antibiotic–ntimycotic solution (Thermo Fisher Scientific, USA) to a final concentration of 1%) were added. The tumor fragment was transferred to a 60 mm Petri dish (SPL, South Korea) and mechanically minced using a #10 medical scalpel (Apexmed, India) into ~1– mm3 pieces. The resulting suspension was transferred to a 15 mL test tube and precipitated in a benchtop centrifuge (Eppendorf) for 5 min (100 g, 24°C). The supernatant was decanted, followed by addition of 2 mL of a warm handling medium containing a mixture of enzymes: 0.5 mg DNase I (Sigma, USA), collagenase types I and IV (Merck, USA) 1 mg of each, and 2 mg hyaluronidase (Microgen, Russia). The tube with the tissue fragments was placed on a rotating platform (Biosan, Latvia) and incubated at 7 rpm in a CO_2_ incubator at 37°C and 8% CO_2_ for 40 min. After incubation, the solution with cells was gently mixed 25–50 times using a serological pipette to break up aggregates until a homogeneous suspension was obtained. The cell suspension was successively filtered through cell sieves with pore sizes of 100, 70, and 40 μm, with the used sieve being additionally washed each time with 2 mL of the handling medium. The cells were precipitated in a benchtop centrifuge (Eppendorf) for 15 min (300 g, 24°C). The cell pellet was treated with 1 mL of ACK buffer (150 mM ammonium chloride; 10 mM potassium bicarbonate; 0.1 mM EDTA-Na2) for 1 min to lyse erythrocytes, when necessary. The reaction was stopped with 2 mL of the handling medium. The cells were pelleted in a benchtop centrifuge (Eppendorf) for 7 min (300 g, 24°C). The supernatant was decanted; the cell pellet was resuspended in 2 mL of the culture medium (DMEM advanced 90% (Thermo Fisher Scientific), 10% HyClone bovine fetal serum (Cytiva, USA), L-alanyl-L-glutamine (Yeasen, USA) up to 2 mM, antibiotic–antimycotic (Thermo Fisher Scientific) up to 1%); and the number of viable cells was estimated by the trypan blue dye exclusion method using a CellDrop FL automated cell counter (DeNovix, USA). An aliquot of the prepared cells was stained, followed by cytometric analysis. The remaining cells were cryopreserved in the CryoMed-M medium (PanEco) according to the manufacturer’s instruction.

**Quantification of B lymphocytes by flow cytofluorometry**

Dissociated tumor cells (2 × 106) were precipitated in a benchtop centrifuge (Eppendorf) for 5 min (350 g, 4°C). The medium was decanted, and the cell pellet was resuspended in 100 μL of phosphate-buffered saline containing 0.5% BSA and 2 mM EDTA. Staining was performed using a mouse monoclonal (clone 2D1) anti-human common leukocyte antigen CD45 antibody conjugated with APC-Cy7 (Sony, USA) at a 1 : 300 dilution and a mouse monoclonal (clone HIB19) anti- human B lymphocyte antigen CD19 antibody conjugated with PE-Cy7 (BioLegend, USA) at a 1 : 1,000 dilution. Incubation with antibodies was performed in the dark at 4°C for 30 min. To identify dead cells, a SYTOX Green dye (BioLegend, USA) was added at a 1 : 3,000 dilution and incubated in the dark at 4°C for more than 15 min. The sample was then washed three times with 500 μL of phosphate-buffered saline containing 2 mM EDTA and resuspended in 100 μL for staining analysis on an ACEA Novocyte flow cytometer (ACEA Biosciences, USA). Statistical data processing was performed using Python tools (seaborn, pandas). The median was estimated in each study group; the decision on the reliability of the differences between analyzed samples was made based on the nonparametric Mann–Whitney U-test.

## RESULTS AND DISCUSSION

**Fig. 1:**
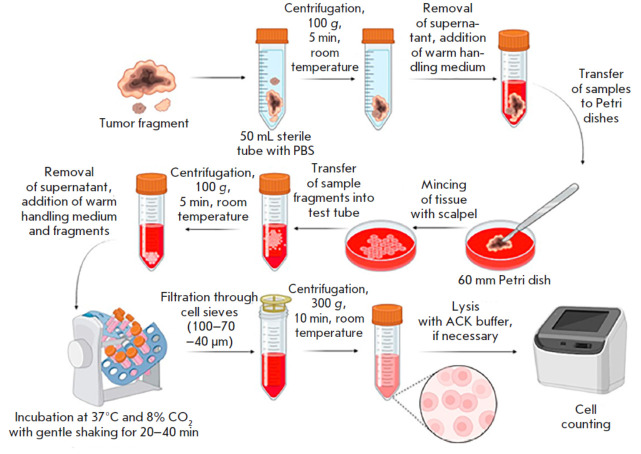
Tumor tissue dissociation workflow

The study included 50 patients with malignancies of various nosologies: CRC (n = 31), NSCLC (n = 13), and CCRCC (n = 6). Cells were isolated from tumor material according to the protocol in [18] with some modifications. The final workflow for preparing a homogeneous cell suspension included mechanical mincing of the sample, dissociation of tissue fragments with a mixture of enzymes, and three successive stages of filtration through cell sieves with pore sizes of 100, 70, and 40 μm (*Fig. 1*).

Mechanical dissociation involves mincing of a tumor fragment into small pieces with a medical scalpel to increase the area of tissue contact with enzymes at the next stage. Enzymatic dissociation by enzymes possessing collagenase, hyaluronidase, and DNase activities is used to destroy the extracellular matrix and prepare a tumor cell suspension. The type of enzymes used for dissociation can significantly affect the quantitative yield and viability of the resulting cells. In the course of the study, we selected an optimal enzyme combination involving hyaluronidase, DNase I, collagenase types 1 and 4 (1.0, 0.25, 0.5, and 0.5 mg/mL, respectively; see Experimental section), the use of which provided effective destruction of cell contacts. This combination reduced the duration of the enzymatic dissociation stage to 30–40 min and increased the yield of viable cells to 80% or more. Filtration of the resulting suspension is necessary for further disaggregation of the cell sample. In this case, the use of a 100 μm cell sieve enabled effective removal of the large cell conglomerates and fat fraction and facilitated further filtration of the sample through 70 and 40 μm sieves to produce a homogeneous cell suspension. Thus, the use of a combination of mechanical and enzymatic dissociation of tumor tissue yielded a homogeneous cell suspension with a high viability level of 88%, on average.

**Fig. 2:**
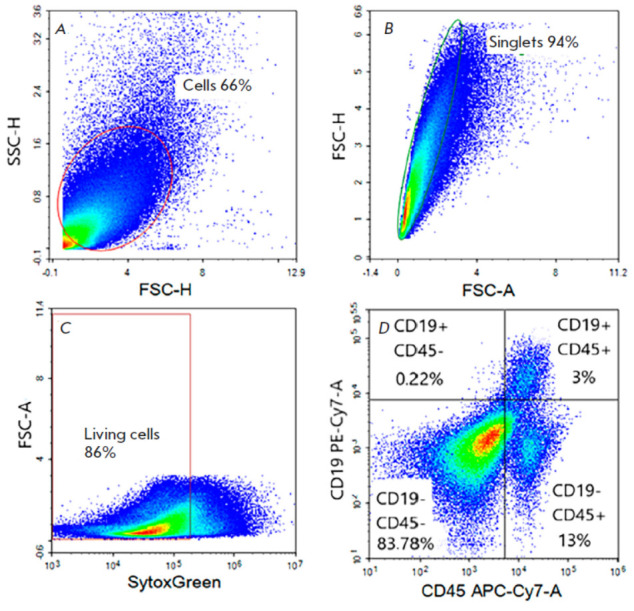
Sequential gating strategy for assessing the fraction of tumor-infiltrating B cells. (*A*) The oval area denotes cells assigned to the tumor cell population based on lateral and forward light scattering data. (*B*) The oval area highlights single cells. (*C*) The rectangular area denotes the living cell population. (*D*) The image is divided into quadrants that are related to the CD45–/CD19–, CD45–/CD19+, CD45+/CD19–, and CD45+/CD19+ cell subsets. The CD45+/ CD19+ cell subset corresponds to B cells

To identify and quantify the B cell population, reflecting the degree of tumor tissue infiltration, all samples were analyzed by flow cytometry (*Fig. 2*). Staining was performed using monoclonal anti-human common leukocyte antigen CD45 antibodies conjugated with APC-Cy7 (CD45 APC-Cy7) and anti-human B lymphocyte antigen CD19 antibodies conjugated with PE-Cy7 (CD19 PE-Cy7). At the first step, a tumor cell population was identified in the forward scatter (FSC) versus side scatter (SSC) dot plot (*Fig. 2A*). Then, single cells were separated from cell aggregates that could be the source of redundant fluorescence signals in further quantification of B cells (*Fig. 2B*). At the second stage, living cells were gated among single cells (*Fig. 2C*) by staining with a fluorescent dye, SytoxGreen, that has high affinity for nucleic acids. The dye penetrates only into cells with damaged plasma membranes, so it is used to assess cell viability. Then, four subsets were identified among the living cells based on staining with specific antibodies to the surface antigens CD45 and CD19, where the gate with double positive staining corresponded to B cells (*Fig. 2D*).

**Fig. 3:**
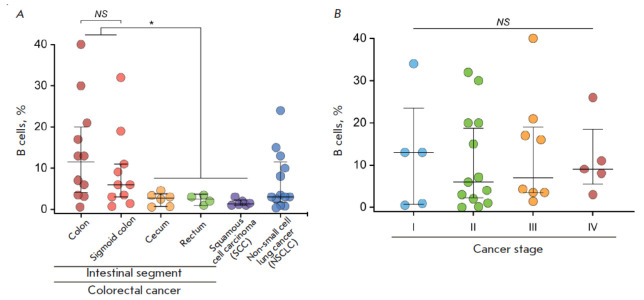
(*A*) Quantification of tumor-infiltrating B cells in tumor material of various nosologies. (*B*) Abundance of tumor-infiltrating B cells in colon cancer samples, depending on the stage. Statistical analysis was performed using the nonparametric Mann–Whitney U test. *P  < 0.05; NS – no significant difference

The cell samples of all studied nosologies were analyzed in the same way. The content of CD19+ B cells varied significantly, from 0.4 to 40% (*Fig. 3A*).

The highest percentage of TIBs was observed in patients with colon and sigmoid colon cancer; the group median was 11.5 and 6%, respectively (*p* < 0.05). In the other groups (cecal and rectal cancer, lung cancer), the median B cell fraction was about 3%. The lowest B cell fraction was observed in kidney cancer, with the median amounting to 1%.

The interquartile range of the TIB fraction was 4–20% for colon cancer, 3–11% for sigmoid colon cancer, 0.7–3.7% for cecal cancer, 0.9–3.6% for rectal cancer, 1.0–2.3% for kidney cancer, and 1.8–12% for lung cancer.

The abundance of TIBs in colon cancer samples was analyzed based on the disease stage (*Fig. 3B*).

Our findings did not reveal statistically significant differences in the percentage of TIBs at different stages of the tumors.

**Table 1:** Association between B cell content and clinical and morphological parameters in colorectal cancer

Parameter		n	CD19+ B cells, %		
			Median	Quartiles 25–75%	p
Age	≤ 63	15	7.0	3.5–15.0	0.428
	< 63	16	3.3	2.1–14.5	
Gender	Male	14	7.0	3.5–20.0	0.135
	Female	17	3.5	2.3–8.5	
Stage	I–II	18	6.0	1.8–16.3	0.805
	III–IV	13	3.5	2.8–12.0	
Grade (G)	G1	4	3.8	2.1–9.5	0.175
	G2–G3	27	15.0	6.0–20.0	
Tumor size (T)	T1–T2	20	1.9	0.2–3.1	0.019*
	T3–T4	11	6.0	3.0–16.0	
Nodal status (N)	N0	29	5.15	2.0–13.8	0.707
	N1	2	4.0	3.0–16.0	
Metastasis (M)	M0	28	4.3	2.3–15.5	0.727
	M1	3	6.0	4.0–8.0	
Location	Large intestine	27	6.0	2.5–16.0	0.255
	Rectum	4	3.25	2.3–3.9	
Large intestine segment	Left	14	5.15	1.7–9.3	0.296
	Right	13	8.0	2.8–18.5	

A generalized analysis of the B lymphocyte content in CRC and the clinical and morphological characteristics of the disease are presented in *Table 1*.

There is a direct correlation between the B cell content and the tumor size; namely, larger tumors are characterized by a higher content of B cells. It is also worth noting that poorly differentiated tumors are characterized by high B cell contents, but that these results do not reach statistical significance.

## CONCLUSION

According to 2020 data, some 1.9 million new cases of colorectal cancer were recorded around the world. Some estimates show that the annual increase in Russia stands at about 50,000 new cases. Colorectal cancer is detected quite late, so the mortality rate attendant to it is rather high and can reach 40% within a year from the time of tumor diagnosis [19], and, according to the World Health Organization, it is the second leading cause of cancer death in the world [20]. Given this, the search for therapeutically significant tumor-specific antigens and/or therapeutic antibodies to these types of cancers is a critical undertaking.

The data obtained in this study deepen our knowledge about the abundance of TIBs in various nosological forms of cancer. We have found that colon cancer is characterized by the highest percentage of TIBs. The material for investigation can be collected regardless of the disease stage, because we have not identified reliable differences in the abundance of B cells at different stages of tumor progression. However, there are conflicting data to the effect that the content of TIBs in colorectal cancer depends on the stage of tumor development [21]. The number of intratumoral B cells is also known to inversely correlate with the stage of lung cancer [22].

From a fundamental point of view, profound profiling of TIBs adds to our knowledge of immune response patterns to cancer cells and will open up new opportunities in the search for potential markers of malignant transformation. From a practical point of view, tumor-infiltrating B cells may be used to create antibody libraries for further development of CAR-T therapy and other personalized therapy approaches.

## Abbreviations

CRC: colorectal cancer
NSCLC: non-small cell lung cancer
CCRCC: clear cell renal cell carcinoma
TIBs: tumor-infiltrating B cells
